# Development and validation of brain-derived neurotrophic factor measurement in human urine samples as a non-invasive effect biomarker

**DOI:** 10.3389/fnmol.2022.1075613

**Published:** 2023-01-12

**Authors:** Alicia Olivas-Martinez, Beatriz Suarez, Elena Salamanca-Fernandez, Iris Reina-Perez, Andrea Rodriguez-Carrillo, Vicente Mustieles, Nicolás Olea, Carmen Freire, Mariana F. Fernández

**Affiliations:** ^1^Centre for Biomedical Research (CIBM), University of Granada, Granada, Spain; ^2^Instituto de Investigación Biosanitaria de Granada, Granada, Spain; ^3^Department of Radiology and Physical Medicine, School of Medicine, University of Granada, Granada, Spain; ^4^Consortium for Biomedical Research in Epidemiology and Public Health, Madrid, Spain

**Keywords:** BDNF, validation, urine, effect biomarker, human health

## Abstract

**Background:**

Brain-derived neurotrophic factor (BDNF), a neurotrophic growth factor mainly expressed in the brain, has been proposed as a potential effect biomarker; that is, as a measurable biomarker whose values could be associated with several diseases, including neurological impairments. The European Human Biomonitoring Initiative (HBM4EU) has also recognized effect biomarkers as a useful tool for establishing link between exposure to environmental pollutants and human health. Despite the well-establish protocol for measuring serum BDNF, there is a need to validate its assessment in urine, a non-invasive sample that can be easily repeated over time. The aim of this study was to develop, standardize and validate a methodology to quantify BDNF protein levels in urine samples before its implementation in biomonitoring studies.

**Methods:**

Different experimental conditions and non-competitive commercial enzyme-linked immunosorbent assay (ELISA) kits were tested to determine the optimal analytical procedure, trying to minimize the shortcomings of ELISA kits. The fine-tune protocol was validated in a pilot study using both upon awakening (*n* = 150) and prior to sleeping (*n* = 106) urine samples from the same Spanish adolescent males in a well-characterized study population (the Spanish INMA-Granada cohort).

**Results:**

The best results were obtained in 0.6 ml of urine after the acidification and extraction (pre-concentration) of samples. The highest reproducibility was obtained with the ELISA kit from Raybiotech. Urinary BDNF concentrations of adolescent males were within the previously reported range (morning = 0.047–6.801 ng/ml and night = 0.047–7.404 ng/ml). Urinary BDNF levels in the awakening and pre-sleep samples did not follow a normal distribution and were not correlated.

**Conclusion:**

The developed methodology offers good sensitivity and reproducibility. Having reliable markers in urine may facilitate both diagnosis and monitoring possible diseases (and treatment). Further studies are needed to implement urinary BDNF in biomonitoring studies to further elucidate its usefulness and biological significance for neurological impairments.

## 1. Introduction

Neurotrophins are a family of proteins synthesized as precursors (pro-neurotrophins) and secreted to the extracellular space as mature proteins with different physiological functions (Gonzalez et al., 2016; Russo et al., 2017). These regulatory factors largely participate in processes involved in the synaptic plasticity, neurogenesis, and maintenance of the structural and functional integrity of the brain and in neuroprotection of the nervous system (Antunes-Lopes et al., 2011; Koven and Collins, 2014; Chen J. et al., 2016; Russo et al., 2017). The most relevant proteins of this family include brain-derived neurotrophic factor (BDNF), nerve growth factor, and neurotrophins 3 and 4 (Gonzalez et al., 2016; Russo et al., 2017).

The growth factor BDNF is expressed throughout the brain, particularly in the hippocampus and prefrontal cortex (Savitz et al., 2006; Lang et al., 2007; Sen et al., 2008). Its activity is mediated by different receptors, with the precursor form (pro-BDNF) having greater affinity for the p75 receptor, triggering apoptosis, and the mature form (m-BDNF) having greater affinity for the tropomyosin-related kinase B receptor (TrkB), being involved in neuronal survival and nervous system morphogenesis (Sen et al., 2008; Chen W. et al., 2016; Gonzalez et al., 2016; Russo et al., 2017). Brain-derived neurotrophic factor is crucial for neuronal development during embryonic and fetal stages and for brain growth, differentiation, and survival during childhood and adulthood (Frias et al., 2011; Jiang et al., 2014; Koven and Collins, 2014), exerting an influence on learning and memory among other cognitive functions (de Coelho et al., 2013). Brain-derived neurotrophic factor circulating in the blood originates in the brain (Collins and Koven, 2014). Once in this peripheral pathway, BDNF may have the ability to be store in platelets, released into circulating plasma by agonist stimulation, transported to the kidney and filtered by nephrons and, finally, excreted in urine (Collins and Koven, 2014). Peripheral blood BDNF levels are considered to provide an optimal estimate of BDNF concentration in the brain, given that it can cross the blood–brain barrier in a bidirectional manner (Koven and Collins, 2014). Circulating levels of BDNF are known to vary according to the age, sex, and physical activity of individuals and the time of day (Lommatzsch et al., 2005; Piccinni et al., 2008; Pal et al., 2014; Perera et al., 2015; Smith et al., 2015; Buchman et al., 2016; Zhao et al., 2017; Ledreux et al., 2019; de Azevedo et al., 2020; Mustieles et al., 2022; Rodríguez-Carrillo et al., 2022a,b). Reduced serum BDNF concentrations were recently described in patients with neurodevelopmental disorders or neurodegenerative diseases (Alzheimer, Parkinson, and Huntington’s), suggesting a possible role for this neurotrophin as an effect biomarker in these diseases (Ciammola et al., 2007; Wang et al., 2015; Amidfar et al., 2020; Gutierrez et al., 2020; Huang et al., 2021; Yi et al., 2021). This neurotrophic factor is also expressed in non-neuronal tissues such as liver, muscle, bladder, kidney, and cardiovascular tissues (Lommatzsch et al., 2005; Noble et al., 2011; Koven and Collins, 2014; Chen et al., 2017; Russo et al., 2017), and its role as a biomarker has been investigated in cases of overactive bladder, enuresis, and benign prostatic hyperplasia (Antunes-Lopes et al., 2013; Alkis et al., 2017; Wang et al., 2017; Peyronnet et al., 2019).

An effect biomarker is defined as a biochemical, physiologic, behavioral, or other alteration (or change) measurable in an organism that, depending on the magnitude, can be recognized as associated with an established or possible health impairment or disease (National Research Council, 2006). Within the European Human Biomonitoring Initiative (HBM4EU), effect biomarkers have been recognized a useful tool to establishing dose–response relationships to environmental pollutants exposure, and even to identify some of their mechanisms of action. By providing a link between exposure, internal dose and health impairment, they could be extremely useful in human biomonitoring and chemical risk assessment studies (Mustieles et al., 2020).

Epidemiological evidence has recently emerged of an association between exposure to different neurotoxic environmental chemicals {e.g., polycyclic aromatic hydrocarbons [PAHs] (Perera et al., 2015), bisphenol A [BPA] (Mustieles et al., 2020), non-persistent pesticides (Rodríguez-Carrillo et al., 2022a), and heavy metals (Rodríguez-Carrillo et al., 2022b)} and alterations in serum BDNF protein levels and/or BDNF gene expression. In spite of the well-establish protocol for measuring BDNF in this biological matrix, there is a high interest in validating the assessment of BDNF in urine, due to the non-invasive characteristics of this biological sample and the fact that its collection can be easily repeated over time. In this context, urinary BDNF concentrations have recently proposed as effect biomarker for the diagnosis of lower urinary tract neurogenic dysfunction or aberrant neurodevelopment (Magalhães et al., 2017; Ece et al., 2019; Richard et al., 2020), and even for prediction of treatment response (Jiang et al., 2014). The determination of BDNF in human urine could also be used as a “potential” effect biomarker in human biomonitoring studies that attempt to establish the relationship between exposure to pollutants of interest and human health, such as those that seek to elucidate the relationship between some environmental exposures and neurodevelopmental disorders (Perera et al., 2015; Mustieles et al., 2020, 2022; Rodríguez-Carrillo et al., 2022a,b).

Despite the attraction of urine as a non-invasive matrix, it is not known how much BDNF is typically cleared through urinary, nor its ratio when compared to other biological fluids (i.e., serum, plasma, cerebrospinal fluid, etc.), and when or what time a peak level of BDNF in the urine is reached.

The few studies that have assessed BDNF concentrations in urine samples (Antunes-Lopes et al., 2013; Collins and Koven, 2014; Russo et al., 2017; Ece et al., 2019; Peyronnet et al., 2019), all of them used the enzyme-linked immunosorbent assay (ELISA). The ELISA technique is widely employed methodology to quantify proteins in biological samples (Sakamoto et al., 2018). Its use, like any tool, has advantages and disadvantages. Among the advantages are: (i) the simplicity of the technique, (ii) its high specificity and sensitivity as it is based on antigen–antibody reactions, (ii) its high efficiency by allowing the simultaneous analysis of a large number of samples, (iv) not requiring organic or radioactive substances, being able to be defined as a safe and environmentally friendly procedure, and (v) being a cheap and cost-effective assay. Among the disadvantages: (i) instability of the antibodies, (ii) insufficient blocking of the immobilized antigen that could interfere with the results obtained, and (iii) the need for refrigeration, and possible affectation of temperature changes during transport, storage and assay (Sakamoto et al., 2018).

The assessment of urinary BDNF proved to be methodologically challenging, when it was decided to perform within HBM4EU, as no standardized protocol was found in the literature, nor was it possible to replicate previously described protocols to measure BDNF in this matrix (Collins and Koven, 2014; Koven and Collins, 2014; Polacchini et al., 2015; Pennycuff et al., 2017). Therefore, the aim of this study, conducted within the European Human Biomonitoring Initiative (HBM4EU), was to fine-tune and validate a methodology to quantify BDNF protein in human urine samples, establishing the most appropriate parameters of sensitivity, reproducibility, range values, and intra-and inter-assay variations and following the corresponding quality assurance and quality control (QA/QC) procedures (HBM4EU - science and policy for a health future, 2016). The protocol resulting from this study was tested in a sample of male adolescents from the Spanish INMA (Environment and Childhood)-Granada birth cohort.

## 2. Materials and methods

### 2.1. Reagents

Extraction of urine samples used trifluoroacetic acid (TFA), methanol, and acetonitrile purchased from Sigma-Aldrich (Sigma-Aldrich, St. Louis, MO, United States), and Strata-XL 60 mg cartridges (#8B-S043-UBJ) from Phenomenex (Phenomex Inc., Germany). Water (18.2 MΩ cm) was purified using an in-house Milli-Q system (Merck Millipore, France). Enzyme-linked immunosorbent assay (ELISA) kits used were Quantikine^®^ Total BDNF (cat# DBTN00 R&D System Inc., Minneapolis, MN, United States), Human BDNF ELISA Kit catalog n°. E-EL-H0010 (Elabscience, Houston, TX, United States), BDNF Human ELISA kit cat# EK-033-22 (Phoenix Pharmaceuticals Inc., Burlingame, CA, United States), BDNF Human ELISA cat# orb50004 (Biorbyt, Explore Bioreagent, United Kingdom), Human ELISA BDNF cat# ab212166 and cat# ab999789 (Abcam, Cambridge, MA, United States), and RayBio^®^ ELISA kit cat# ELH-BDNF (Raybiotech, Norcross, GA, United States). All ELISA kits used were sandwich and non-competitive type assays. Supplementary Table 1 summarizes the main characteristic of all of them.

### 2.2. INMA-Granada study

The study sample comprised 15-17-year-old adolescent males from the INMA-Granada cohort. The INMA Project is a multicenter population-based birth cohort study designed to investigate the effects of exposure to environmental chemicals during pregnancy on fetal and childhood development in seven geographical areas of Spain (Guxens et al., 2012). The INMA-Granada cohort recruited 668 mother-boy pairs between 2000 and 2002 at the *Hospital Universitario San Cecilio* (HUSC) (Granada, Spain) with the initial aim of evaluating the relationship between prenatal exposure to endocrine disrupting chemicals and male urogenital malformations (Fernandez et al., 2007). Follow-ups have been performed when the children were 4–5 (2005–2006, *n* = 220), 9–11 (2010–2012, *n* = 300), and 15–17 (2017–2019; *n* = 155) years old (Pérez-Lobato et al., 2015; Freire et al., 2018; Castiello et al., 2020; Mustieles et al., 2022). The principles of the Declaration of Helsinki (World Medical Association, 2013) were followed, and both the initial study and all follow-ups were approved by the Biomedical Research Ethics Committee of Granada. An informed consent form was signed by the parents of all participants for all follow-ups.

Urine samples were obtained during the 2017–2019 follow-up of the INMA-Granada cohort (Castiello et al., 2020; Suárez et al., 2021). Out of 155 followed-up adolescents, urine upon awakening was collected from 150, and 106 of these also provided a prior to sleeping sample (Castiello et al., 2020; Suárez et al., 2021). Participants self-collected the urine prior to sleeping of the previous day and the urine upon awakening of the day of their follow-up visit at the hospital. Participants kept both samples at 4°C until their arrival at the hospital, where were aliquoted in several tubes (1–2 ml) and stored at −80°C at the Center for Biomedical Research (CIBM) of the University of Granada (Spain).

All urine samples (*n* = 256) were analyzed in duplicate and in multiple assays to calculate intra-and inter-variations in BDNF values as well as the mean value. In addition, urinary creatinine concentrations (mg/dL) were measured at the Scientific and Technical Platform of the *Instituto de Investigación Biosanitaria de Granada* (ibs.Granada) to account for urine dilution. Urinary BDNF concentrations were normalized by creatinine concentrations and expressed as ng BDNF/mg creatinine (BDNF/Cr).

### 2.3. Statistical analysis

Descriptive statistical parameters were calculated, including arithmetic and geometric mean, standard deviation (SD), median, 25th and 75th percentiles, and minimum and maximum BDNF concentrations. Urinary BDNF concentrations below the assay limit of detection (LOD) were assigned a value of LOD/√2. Normality of the distribution of urinary BDNF concentrations was evaluated with the Kolmogorov–Smirnov test. Spearman’s correlation test was performed to assess the relationship between morning and bedtime urinary BDNF concentrations. SPSS version 28 (IBM SPSS, Armonk, NY, United States) was used for statistical analyses.

## 3. Results and discussion

### 3.1. Development and validation of the methodology to quantify BDNF in human urine samples

The following experimental conditions were tested for the fine-tuning and validation of a methodology to quantify BDNF protein in human urine samples, in accordance with an appropriate QA/QC process: (i) analysis of urine samples with and without pre-treatment (acidifying, extracting and/or lyophilizing of biological samples); (ii) dilution (or not) of samples; (iii) testing of different sample volumes (range 0.1–0.8 ml); and (iv) selection of commercial ELISA kits, based on the literature (Supplementary Table 2), because ELISA techniques have demonstrated good sensitivity and specificity for the evaluation of BDNF levels (Sakamoto et al., 2018). The Emax^®^ ImmunoAssay System ELISA kit (Promega Corporation, Madison, WI, United States), one of the kits most commonly used to determine BDNF in urine samples (Pinto et al., 2010; Antunes-Lopes et al., 2013, 2017; Jiang et al., 2014; Morizawa et al., 2019), has been discontinued. Other ELISA kits previously used for this purpose could not be used because samples were always below their LOD, with or without pretreatment, including the Quantikine^®^ Total BDNF and Human BDNF ELISA kits (cat# DBNT00, cat# E-EL-H0010, and cat# EK-033-22), supplied by R&D System, Elabscience and Phoenix Pharmaceuticals, respectively (Polacchini et al., 2015; Pennycuff et al., 2017; Rada et al., 2020). In addition, very low reproducibility was found when the BDNF Human ELISA (cat# orb50004) from Biobyrt was applied in accordance with the protocol described by Russo et al. (2017).

Human ELISA BDNF cat# ab212166, a new kit from Abcam, was also tested, but the reagent used to dilute and/or reconstitute urine samples was found to interfere with the optical density measurement and the BDNF concentration. BDNF assessment was then performed with ELISA kit cat# ab999789, also supplied by Abcam (Collins and Koven, 2014; Koven and Collins, 2014; Ozdemir et al., 2016), which uses two diluents, one (A) for serum or plasma samples and another (B) for cell culture supernatant samples. In this validation study, assays were performed with both diluents, but the best results were obtained when dry human urine sample extracts, obtained by a previous treatment, were reconstituted with diluent B. Unfortunately, however, this kit is no longer produced, and a new search was undertaken for a kit with similar characteristics to those of Abcam cat# ab999789. The RayBio^®^ ELISA kit cat# ELH-BDNF, supplied by Raybiotech (Norcross, GA, United States) and used by Peyronnet et al. (2019), was then tested in accordance with the manufacturer’s recommendations. The results obtained showed this kit to be optimal for the assessment of BDNF in human urine samples.

Difficulties found with the previous ELISA kits were related either to their lack of specificity for urine samples, to their low sensitivity and reproducibility, or to the percentage of values below the LOD (R&D System cat# DBNT00, Elabscience cat# E-EL-H0010, Phoenix Pharmaceuticals cat# EK-033-22, and Abcam cat# ab212166). Some of these ELISA kits, however, have been shown to provide good results in serum or plasma samples (Piccinni et al., 2008; Polacchini et al., 2015). After several assays under the experimental conditions described above, the optimal method to quantify the total BDNF concentration in human urine samples was found to be the pre-ELISA protocol proposed by Collins and Koven (2014) and Koven and Collins (2014) with minor modifications. In addition, to achieve higher sensitivity, the sample was pre-treated (acidification plus extraction) which improved the results. Thus, first, urine samples were acidified and extracted by adding 0.3 ml of 1.5% TFA to 0.6 ml of urine, vortexing, and then centrifuging for 15 min (4,000 rpm) at 4°C. The supernatant was loaded into a Strata-XL 60 mg cartridge previously equilibrated with 1 ml methanol (MeOH) and 1 ml distilled water and allowed to drain. The column was then washed with 0.1% TFA (1 ml) and vacuum aspiration was applied for 10 min to remove any residual fluids. Finally, the cartridge was eluted with 3 ml of acetonitrile (80%), and the resulting eluent was evaporated to complete dryness under a nitrogen stream. The resulting dried extract was reconstituted with 220 μl of diluent B in the RayBio^®^ ELISA kit and analyzed according to the manufacturer’s instructions (Supplementary Figure 1). The same urine samples were always used for all ELISA assays. In the fine-tuning of the protocol, some of the ELISA drawbacks were also taken into account, e.g., maintaining room temperature (avoiding changes and variability during the assay), ensuring that the cold chain was not broken, using the same ELISA lot number for all samples, and ensuring blocking of the immobilized antigen controlling incubation times. For more details on the methodology, see Supplementary Figure 1.

The urinary BDNF concentrations obtained were within previously reported ranges (Collins and Koven, 2014; Koven and Collins, 2014; Ozdemir et al., 2016), the within-assay coefficient of variation (CV) was <5%, and the between-assay CV was <15%. The sensitivity of the assay was <80 pg./ml and the detection range 0.06–16 ng/ml.

### 3.2. Quality assurance and quality control (QA/QC) assessment

All calibration curve standards, blanks (zero standards), and human urine samples were analyzed in duplicate in the 96-well microplate in the selected RayBio^®^ ELISA kit (cat# ELH-BDNF). Reagents and standards were prepared according to the manufacturer’s instructions, diluting diluent B five-fold in Milli-Q distilled water (15 ml in 60 ml of water) and diluting the buffer concentrate 20-fold in Milli-Q distilled water (20 ml of 20X buffer in 380 ml of water) to obtain 400 ml of 1X wash buffer. The 1X diluent B was used to prepare the antibody detection reagent (200 μl of antibody detection concentrate and 16 ml of 1X diluent B) and HRP-streptavidin reagent (75 μl of HRP-streptavidin concentrate and 15 ml of 1X diluent B).

A standard curve was prepared from the stock solution (400 ng/ml) by mixing 720 μl of assay diluent B with BDNF powder from the manufacturer (Table 1); the curve had seven calibration points. Assay diluent B 1X served as a blank.

**Table 1:** Preparation of the standard curve.

Standard number	Preparation
# 1 (16 ng/ml)	40 μl stock solution + 960 μl assay diluent B
# 2 (6.4 ng/ml)	200 μl Std. 1 + 300 μl assay diluent B
# 3 (2.56 ng/ml)	200 μl Std. 2 + 300 μl assay diluent B
# 4 (1.02 ng/ml)	200 μl Std. 3 + 300 μl assay diluent B
# 5 (0.41 ng/ml)	200 μl Std. 4 + 300 μl assay diluent B
# 6 (0.16 ng/ml)	200 μl Std. 5 + 300 μl assay diluent B
# 7 (0.06 ng/ml)	200 μl Std. 6 + 300 μl assay diluent B

Std.: Standard.

**Figure 1:**
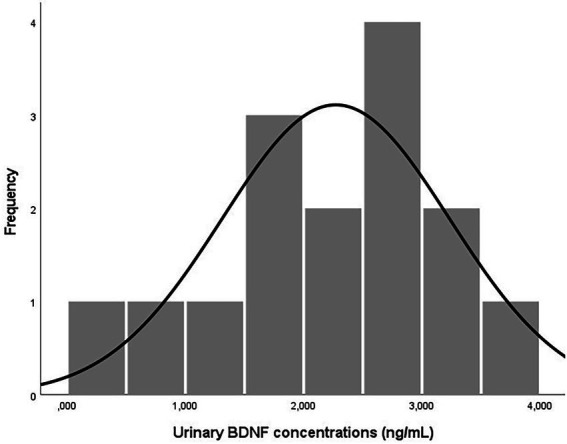
Distribution of BDNF concentrations in human urine samples (*n* = 15).

For QC assays, 15 anonymized human urine samples were selected from routine hospital analyses and treated according to the protocol developed and described above. Dried urinary extracts were reconstituted with 220 μl of 1X assay diluent B, and 100 μl of standard, blank, or urinary samples was added to each well in the ELISA kit 96-well plate and incubated for 2.5 h at room temperature (RT) with gentle shaking. The solution was then discarded and carefully washed 4 times with 1X Wash solution (300 μl/well). Next, 100 μl of 1X Biotin Antibody solution was added to each well and incubated for 1 h under the above conditions, discarding the solution and performing the washes. After incubation, 100 μl of 1X streptavidin solution was added to each well and incubated for 45 min at RT with gentle shaking, again discarding the solution and repeating the washes. Then, 100 μl of tetramethylbenzidine (TMB) one-step substrate reagent was added to each well and incubated for 30 min at RT under gentle agitation in the dark until color development visualization. Finally, after the addition of 50 μl of Stop solution, the well plate was immediately read at 450 nm using a microplate reader (BioTek HTX, Fisher Scientific, United States; Supplementary Figure 1). Table 2 and Figure 1 exhibit the final concentrations of BDNF (ng/ml) obtained in the selected urine samples.

**Table 2:** Absorbance and BDNF concentrations in duplicate human urine samples.

Sample	Absorbance			Concentration		
	450 nm		Mean ± SD	ng/ml		Mean ± SD
1	0.501	0.484	0.493 ± 0.012	2.902	2.816	2.859 ± 0.061
2	0.354	0.333	0.344 ± 0.015	2.129	2.013	2.071 ± 0.082
3	0.510	0.320	0.415 ± 0.134	2.947	1.941	2.444 ± 0.712
4	0.355	0.731	0.543 ± 0.266	2.135	4.000	3.067 ± 1.319
5	0.330	0.313	0.322 ± 0.012	1.997	1.901	1.949 ± 0.067
6	0.441	0.548	0.495 ± 0.076	2.594	3.136	2.865 ± 0.383
7	0.504	0.498	0.501 ± 0.004	2.917	2.887	2.902 ± 0.021
8	0.143	0.230	0.187 ± 0.062	0.883	1.420	1.152 ± 0.380
9	0.563	0.569	0.566 ± 0.004	3.210	3.239	3.225 ± 0.021
10	0.279	0.288	0.284 ± 0.006	1.708	1.759	1.734 ± 0.037
11	0.716	0.694	0.705 ± 0.016	3.931	3.831	3.881 ± 0.071
12	0.067	0.094	0.081 ± 0.019	0.381	0.563	0.472 ± 0.129
13	0.157	0.096	0.127 ± 0.043	0.972	0.577	0.774 ± 0.280
14	0.517	0.463	0.490 ± 0.038	2.983	2.708	2.845 ± 0.194
15	0.315	0.285	0.300 ± 0.021	1.913	1.742	1.827 ± 0.121
Descriptive statistics for urinary BDNF concentrations (ng/ml)						
Arithmetic mean			2.271	Min.	0.472	
Median			2.444	Max.	3.881	
SD			0.962	Intra-assay CV	4.16%	

BDNF, brain derived neurotrophic factor; CV, coefficient of variation; Min., minimum; Max., maximum; SD, standard deviation.

The results obtained in the QA/QC assay showed urinary BDNF concentrations within the previously reported range (Supplementary Table 2; Collins and Koven, 2014; Koven and Collins, 2014; Kurku et al., 2019).

### 3.3. Application to INMA-Granada urine samples

After developing the optimal method to analyze BDNF concentrations in urine samples, their validity was evaluated as a possible neurological effect biomarker in human biomonitoring studies. Available urine samples from the well-characterized study population (the Spanish INMA-Granada cohort), specifically from male adolescents aged 15–17 years, were used (Pérez-Lobato et al., 2015; Freire et al., 2018; Castiello et al., 2020; Mustieles et al., 2022; Rodríguez-Carrillo et al., 2022a,b). This cohort was also selected because one of its objectives was to determine the effect of exposure to different endocrine-disrupting chemicals (such as non-persistent pesticides) on the neurodevelopment of the participants (Freire et al., 2021; Suárez et al., 2021). All urine samples (*n* = 256) obtained from the adolescent males, both upon awakening (*n* = 150) and before sleep (*n* = 106), were assessed in duplicate. Table 3 displays the urinary BDNF concentrations measured, the mean value, and the mean intra-and inter-assay CVs obtained (4.56 and 11.28%, respectively).

**Table 3:** Urinary BDNF concentrations in adolescent males (15–17 years).

	Samples upon awakening (*n *= 150)		Samples prior to sleeping (*n* = 106)	
	BDNF (ng/ml)	BDNF/Cr (ng/mg)	BDNF (ng/ml)	BDNF/Cr (ng/mg)
Range	0.047–6.801	0.049–15.173	0.047–7.404	0.041–9.975
Arithmetic mean	3.613	2.174	1.886	1.268
SD	1.344	1.494	1.817	1.598
P25	3.014	1.486	0.245	0.189
P50	3.747	1.927	1.495	0.861
P75	4.236	2.511	2.985	1.780
<LOD (%)	1.30		2.83	
Intra-assay CV (%)	4.32		4.82	
Inter-assay CV (%)	11.07		11.44	

BDNF, brain derived neurotrophic factor; Cr, creatinine; CV, coefficient of variation; LOD, limit of detection; SD, standard deviation.

**Figure 2:**
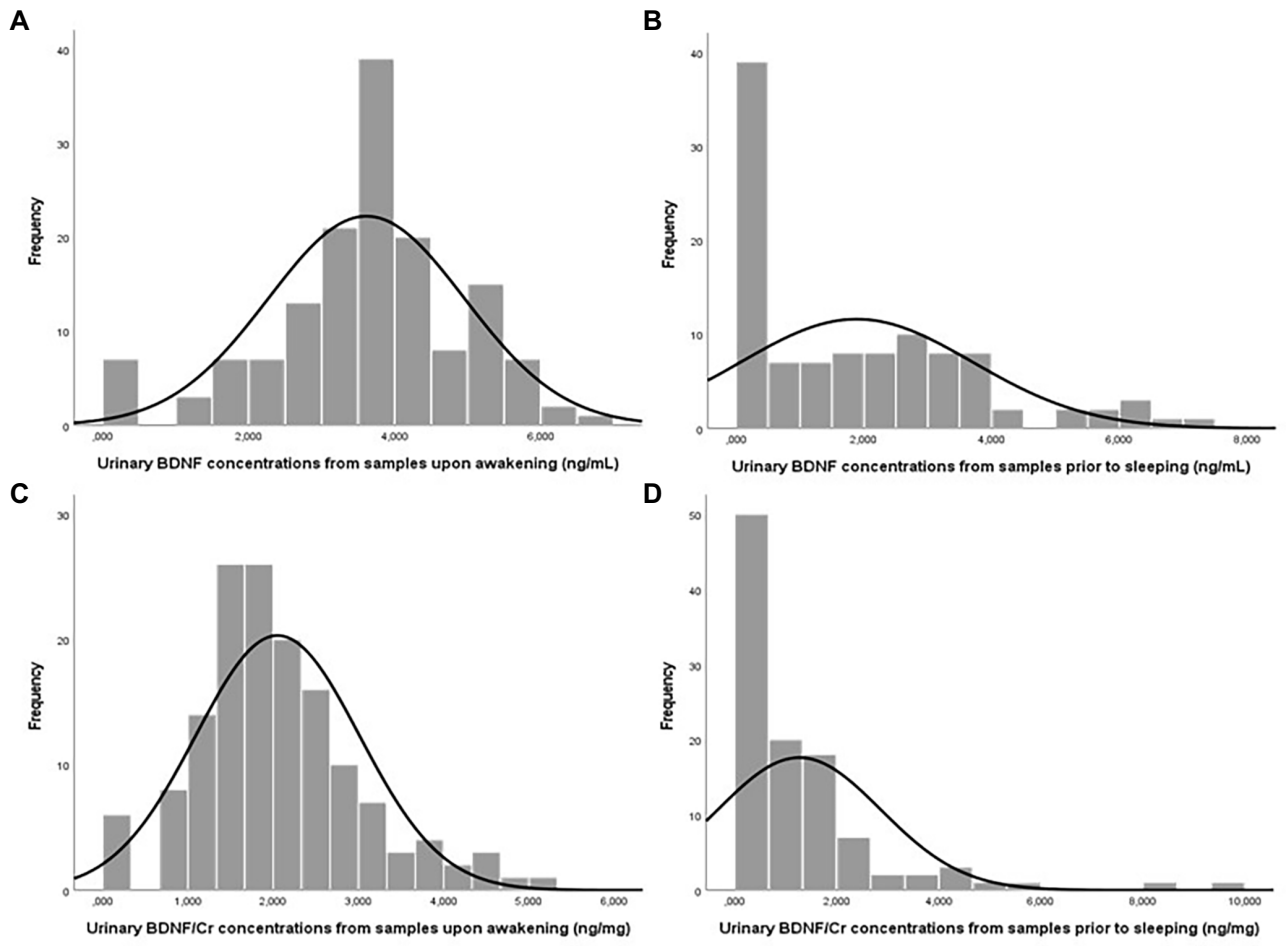
Urinary BDNF (ng/ml) and BDNF/Cr (ng/mg) concentrations in male adolescents: Urine samples upon awakening, *n* = 150 **(A,C)** and samples prior to sleeping, *n* = 106 **(B,D)**.

The detection limit was 0.047 ng/ml, and only a very small number of urine samples (2.1%) were below the LOD, indicating that the protocol offers adequate sensitivity (Table 3). The concentrations of BDNF did not follow a normal distribution in either upon awakening (Kolmogorov–Smirnov test, *D* = 0.101, *p* < 0.001) or pre-sleep samples (*D* = 0.158, *p* < 0.001; Figure 2), and no significant correlation was found between BDNF concentrations of upon awakening and prior to sleeping samples (Spearman correlation coefficient, rho = 0.01, *p* = 0.162, *n* = 106; Supplementary Figure 2). Similar results were obtained when BDNF measurements were adjusted for creatinine concentrations (rho = 0.019, *p* = 0.325, *n* = 106; Supplementary Figure 2).

The urinary BDNF concentrations found among adolescent males were also within the range reported previously in the literature (Supplementary Table 2). For example, two previous studies of BDNF in urine from male and female adolescents (10–15 years; Ozdemir et al., 2016; Kurku et al., 2019) described similar BDNF concentrations to those observed in the present series.

Brain-derived neurotrophic factor concentrations were higher in upon awakening (median = 3.747 ng/ml) versus prior to sleeping (median = 1.495 ng/ml) urine samples, as previously observed in plasma BDNF samples from adult males, although plasma concentrations were found to be more stable throughout the day in adult females (Piccinni et al., 2008). Some authors suggested that this variation could be related to circulating cortisol concentrations, observing a similar pattern for both markers over the day (Weitzman et al., 1971; Piccinni et al., 2008). This proposal was supported by Begliuomini et al. (2008), who described a circadian rhythm in circulating BDNF concentrations and found a positive correlation between plasma BDNF and cortisol concentrations. Although BDNF and cortisol have different expression mechanisms, their secretion and functions in brain physiology are integrated by the dynamics of glucocorticoid receptors, suggesting that they might play a synergistic role in the homeostasis of brain functions (Begliuomini et al., 2008; de Assis and Gasanov, 2019).

Given that serum BDNF concentrations (*n* = 132) were available for the same adolescents from the INMA-Granada cohort (Supplementary material; Rodríguez-Carrillo et al., 2022a,b), these were compared with urinary concentrations obtained in the same adolescents. The concentrations of BDNF were higher in serum samples than samples upon awakening or prior to sleeping (*n* = 92, median = 33.835 ng/ml vs. 3.627 ng/ml and 1.712 ng/ml, respectively). A weak but significant positive correlation was found between serum and urinary BDNF concentrations in samples upon awakening (rho = 0.084, *p* = 0.01, *n* = 132) but not between serum and samples prior to sleeping (rho = 0.007, *p* = 0.702, *n* = 92; Supplementary Figure 3).

It has recently been proposed that the measurement of BDNF concentrations in urine could serve as a biomarker to establish a more accurate clinical diagnosis in some pathologies (such as neurogenic lower urinary tract dysfunction or inappropriate neurodevelopment; Magalhães et al., 2017; Ece et al., 2019; Richard et al., 2020), and even as a potential predictor of response to treatment (Jiang et al., 2014). Several studies have also been able to identify and/or establish BDNF cut-off points in serum and plasma samples for different neurological pathologies or disorders (Kim et al., 2007; Dreimüller et al., 2012; Hong et al., 2014; Rabie et al., 2014; Zhang et al., 2014; Chiou and Huang, 2016; Chiou and Huang, 2019; Lin et al., 2021). The lack of knowledge about the concentration of BDNF excreted in urine of healthy population, or the “cut-off point” that would allow categorizing inappropriate situations, for example, in relation to neurodevelopment, implies further work in this area of knowledge. Our results indicate that BDNF values in urine samples at awakening (fasting sample) correlate well with serum BDNF levels. However, morning values may reach extreme values of an inverted U-shaped metabolism and, therefore, it will be necessary to explore mid-day levels as well. Therefore, future studies are needed to solve these gaps in knowledge. Future animal studies should also attempt to evaluate the serum/plasma and cerebrospinal fluids (CSF) to urine ratio, to help better understand urinary levels, and its proportionality in both general and clinical subjects.

From a clinical point of view, having reliable markers in urine (a non-invasive sample that can be easily repeated over time) may facilitate both diagnosis and monitoring of a possible disease and its treatment. Further studies are also needed to implement urinary BDNF as a biomarker of effect for neurological disease and to understand the biological significance of this protein in urine. The limitations of this study include: (i) the inability to assess possible sex-related differences in urinary BDNF levels; (ii) the lack of precise timing for urine sample collections, adding variability to the results; (iii) the modest sample size; (iv) those related with disadvantages of the ELISA technique [instability of the antibodies, insufficient blocking of the immobilized antigen, and affectation of temperature changes (Sakamoto et al., 2018)]; as well as, (v) the need to ensure the validity of this initial step in future studies, which reproduce the results of the ELISA used, and allow comparison of BDNF levels in urine with the corresponding levels in other biological fluids (serum, CSF, etc.). As strengths, we highlight the development of an accessible and reproducible methodology to measure BDNF in human urine samples that takes numerous variables into account. In addition, unlike serum or plasma, the collection of urine is non-invasive and can be readily repeated over time. Among the advantages of the ELISA technique are: (i) the simplicity of the technique, (ii) its high specificity and sensitivity as it is based on antigen–antibody reactions, (ii) its high efficiency by allowing the simultaneous analysis of a large number of samples, (iv) not requiring organic or radioactive substances, being able to be defined as a safe and environmentally friendly procedure, and (v) being a cheap and cost-effective assay.

## 4. Conclusion

A methodology to quantify BDNF in human urine samples has been developed and validated, demonstrating good sensitivity and reproducibility. Urinary BDNF levels could help to elucidate the relationship between exposure to environmental chemicals or their mixtures and adverse neurological outcomes (Mustieles et al., 2022). Given the less invasive characteristics of urine collection and the potential benefits of a valid effect biomarker, urinary BDNF concentrations should be considered in future biomonitoring studies to strengthen evidence on their usefulness and elucidate their biological significance.

## Data availability statement

The original contributions presented in the study are included in the article/Supplementary material, further inquiries can be directed to the corresponding author.

## Ethics statement

The INMA-Granada cohort study was approved by the Comité Ético de Investigación Provincial de Granada (CEI)/Granada Provincial Research Ethics Committee (CEI). Written informed consent to participate in this study was provided by the participants’ legal guardian/next of kin.

## Author contributions

AO-M and BS: methodology, validation, formal analysis, investigation, conceptualization, and writing – original draft. ES-F, IR-P, AR-C, and VM: investigation, methodology, and writing – review and editing. NO: methodology, research supervision, and writing – review and editing. CF: research supervision, methodology, conceptualization, writing – review and editing, and approval original draft. MF: global idea and research supervision, investigation, methodology, conceptualization, writing – review and editing, approval original draft, and project administration. All authors contributed to the article and approved the submitted version.

## Funding

This study was supported by the European Union’s Horizon 2020 research and innovation program HBM4EU under Grant Agreement #733032, by the Biomedical Research Networking Center-CIBER de Epidemiología y Salud Pública (CIBERESP) of the Institute of Health Carlos III, and by research grants from the Institute of Health Carlos III (SCIII) – supported by the European Regional Development Fund/FEDER (FIS-PI17/01526 and FIS-PI20/01656). The authors are also grateful to the ISCIII for the predoctoral contract granted to AO-M (Grant no. FI21/00236) and the Miguel Servet Type II Program granted to CF (Grant no. CP1121/00014) and to the Spanish Ministry of Education for the predoctoral contract granted to IR-P (Grant no. FPU17/01848). Junta de Andalucía-PAIDI (Spain) is also gratefully acknowledged for funding postdoctoral grants to ES-F. This article is part of the PhD thesis developed by AO-M in the context of the “Clinical Medicine and Public Health Program” of the University of Granada.

## Conflict of interest

The authors declare that the research was conducted in the absence of any commercial or financial relationships that could be construed as a potential conflict of interest.

## Publisher’s note

All claims expressed in this article are solely those of the authors and do not necessarily represent those of their affiliated organizations, or those of the publisher, the editors and the reviewers. Any product that may be evaluated in this article, or claim that may be made by its manufacturer, is not guaranteed or endorsed by the publisher.
