# Supplementary material for: Development and validation of brain-derived neurotrophic factor measurement in human urine samples as a non-invasive effect biomarker
Source: Front Mol Neurosci. 2023 Jan 12;15:1075613. doi: 10.3389/fnmol.2022.1075613 (PMC9878568; doi:10.3389/fnmol.2022.1075613)
Supplement: Supplementary file 1 [file Data_Sheet_1.docx]

Supplementary Material

# 2. Materials and Methods

**2.1 Reagents**

**Supplementary Table 1.** Main characteristics of the ELISA kits used in this study, as declared by the manufacturers.

| **Company** | **R&D Systems** | **Elabscience** | **Phoenix Pharmaceuticals** | **Biobyrt** | **Abcam** | | **Raybiotech** |
| --- | --- | --- | --- | --- | --- | --- | --- |
| **Reference** | **#BDNT00** | **#E-EL-H0010** | **#EK-033-22** | **#orb50004** | **#ab212166** | **#ab99978** | **#ELH-BDNF** |
| **Principle of the assay** | Sandwich ELISA | | | | | | |
| **Sensitivity** | 1.35 pg/mL | 18.75 pg/mL | 7.8 pg/mL | < 15 pg/mL | 2.4 pg/mL | <80 pg/mL | |
| **Range of detection** | 15.6-1000  pg/mL | 31.25-2000 pg/mL | 7.8-500 g/mL | 31.2 – 2000 pg/mL | 15.6 - 1000 pg/ml | 0.066-16 ng/mL | |
| **BDNF standard** | Human recombinant | BDNF standard (type not declared) | Human recombinant | | | | BDNF standard (type not declared) |
| **Coating/capture antibody** | Pre-coated BDNF antibody (not type declared) | | | Pre-coated BDNF antibody (mouse monoclonal) | Pre-coated BDNF antibody  (not type declared) | | |
| **Primary detection antibody** | BDNF antibody (monoclonal) –  HRP conjugated | Biotinylated BDNF antibody (not type declared) | | Biotinylated BDNF antibody (goat polyclonal) | Antibody Cocktail (not type declared) | Biotinylated BDNF antibody (not type declared) | |
| **Type of secondary detection** |  | Streptavidin-HRP conjugated | | Avidin-Biotin-Peroxidase complex |  | Streptavidin-HRP conjugated | |
| **Sample** | Serum, plasma, cell culture supernatant, tissue lysate, human milk and urine | Serum, plasma, tissue homogenate and other biological fluids | Serum, plasma, culture media, tissue homogenate, cerebrospinal fluid, urine or any biological fluid | Serum, plasma, cell culture supernatant, tissue lysate or body fluids | Serum, plasma, cell culture supernatant | | |
| **Processing time** | 3-4 hours | | 5-6 hours | 3-4 hours | 1-2 hours | 4-5 hours | |

BDNF: Brain-derived neurotrophic factor

# 3. Results and discussion

**3.1. Development and validation of the methodology to quantify BDNF in human urine samples**


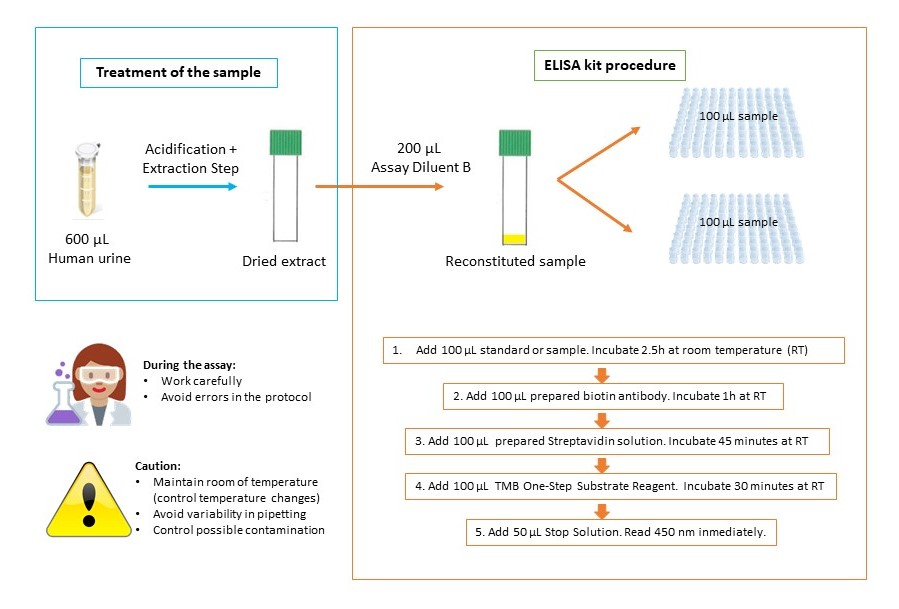


**Supplementary Figure 1.** Flow chart of the methodology to quantify BDNF in human urine samples.

**Supplementary Table 2**. Summary of the scientific literature about BDNF in human urine samples

| **Citation** | **Country** | **Sample size** | **Study population** | **Age (years)** | **Urine sample collection** | **BDNF measurement** | **BDNF concentrations** |
| --- | --- | --- | --- | --- | --- | --- | --- |
| This study | Spain | 150/106 | **Healthy teenagers:**  Male | 15-17 | First morning/Bedtime | **ELISA kit:**  #ELH-BDNF Raybio from Raybiotech | **BDNF (ng/mL):** 3.613 ± 1.344 / 1.886 ± 1.817  **BDNF/Cr (ng/mg):** 2.174 ± 1.494 / 1.268 ± 1.598 |
| Kurku et al., 2019 | Turkey | 30 | **Healthy subjects:**  16 males and 14 females | 12.53 ± 2.24 | Morning (fasting) | **ELISA kit:**  #CK-E10186 from Hangzhou Eastbiopharm Co. | **BDNF (ng/mL):** 0.670 ± 0.271  **BDNF/Cr (ng/mg):** 0.931 ± 0.618 |
| Philippova et al., 2021 | Russia | 20 | **Healthy subjects:**  10 males and 10 females | 44.2 ± 13.6  (range: 25-64) | Voided urine | **ELISA kit:**  Emax®ImmunoAssay System from Promega | **BDNF (ng/ml):** 0.0468 ± 0.0223 |
| Wang et al., 2017 | Wuhan | 32 | **Healthy subjects:**  Males | 50-64 | Voided urine | **ELISA kit:**  Boster Biological Technology | **Total BDNF (ng/mL):**  0.0236 ± 0.0128 |
| Ghoniem et al., 2011 | Weston (USA) | 20 | **Healthy subjects:** Premenopausal females | ≥18 | Midstream urine | **Array of cytokines:**  RayBiotech | **BDNF (densitometric units):** 0.414±0.09 |
| Ozdemir et al., 2016 | Turkey | 30 | **Healthy children:**  8 males and 22 females | 5-15 | Morning | **ELISA kit:**  #ab99978 from Abcam | **BDNF/Cr (ng/mg):**  0.81 ± 0.71 |
| Ece et al., 2019 | Turkey | 29 | **Healthy children** | 6-16 | Between 10.00 and 12.00h a.m. | **ELISA kit:**  Sun Red Biological Technology | **BDNF/Cr (ng/mg):**  1.2x10^-5^ |
| Koven and Collins, 2014 | Lewiston (USA) | 52 | **Healthy subjects:**  20 males and 32 females | 18-22 | Between 11.30 and 13.30 h a.m. | **ELISA kit:**  #ab99978 from Abcam | **BDNF (ng/mL):** 0.6 ± 1.1  (range 0.1-7.9) |
| Rada et al., 2020 | Romania | 50 | **Healthy subjects:**  pre- and post-menopausal females | 49 ± 9.06 | Over a 24 hours period | **ELISA Kit:**  Elabscience | **hBDNF/uCr ratio:**  < 1x10^-7^ (units N.R.) |
| Aisa et al., 2021 | Italy | 30 | **Healthy children** | 2 | First morning urine | **ELISA kit:**  #ERBDNF from Thermo Scientific | **BDNF/Cr (ng/mg):**  0.43 ± 0.4  (range 0.12−1.43) |
| March et al., 2021 | Australia | 15 | **Healthy subjects:**  males | 65  (range: 28– 79) | First-pass urine when they had a desire to void. | **ELISA kit:**  #BEK- 2211 for BDNF, #BEK- 2237 for proBDNF from Biosensis Pty Ltd | **BDNF (ng/mL):** 0.0042 |
| Wang et al., 2014 | Wuhan | 45 | **Healthy subjects:**  Females | <55 | Morning | **ELISA kit:**  Boster Biological Technology | **BDNF/Cr (ng/mg):** 0.00165 (range 0.00128-0.00204) |
| Pennycuff et al., 2017 | Atlanta (GA) | 29 | **Healthy subjects:**  Females | 64 ± 9 | N.R. | **ELISA Kit:**  Phoenix Pharmaceuticals | **BDNF/Cr (ng/mg):** 4.7 ± 5.4 |
| Morizawa et al., 2019 | Japan | 25 | **Healthy children:**  18 males and 7 females | 8-12 | Morning | **ELISA kit:**  Emax®ImmunoAssay System from Promega | **BDNF/Cr ratio:** 1.03  (range 0.0094‐14.80)  (Units N.R.) |
| Jiang et al., 2014 | Jakarta  (Indonesia) | 45 | **Healthy subjects** | 45.96 ± 14.0  (range 19–58) | Voided urine | **ELISA kit:**  Emax®ImmunoAssay System from Promega | **BDNF (ng/mL):** 0.00957 ± 0.00537 |
| Antunes-Lopes et al., 2017 | Porto (Portugal) | 20 | **Healthy subjects:**  Females | 42.7 ± 14.8  (range 25-74) | When subjects experienced a comfortable desire to void. | **ELISA kit:**  Emax®ImmunoAssay System from Promega | **Log BDNF/Cr**: 1.81 ± 0.90 (units N.R.) |
| Jiang et al., 2022 | Taiwan | 25 | **Healthy subjects:**  Females | 60.5 ± 10.6  (range: 41–68) | Self-voided by people when had a full bladder sensation | **Panel kit:**  Milliplex® human cytokine/ chemokine magnetic bead-based (HNDG3MAG-36 K) from Millipore | **BDNF (ng/mL):** 0.00058 ± 0.00015 |
| Collins and Koven, 2014 | Lewiston (USA) | 52 | **Healthy subjects:**  20 males and 32 females | 19.2 ± 1.3 | Between 11.30 and  13.30h a.m. | **ELISA kit:**  #ab99978 from Abcam | **BDNF (ng/mL):** 0.6 ± 1.1 (range 0.1-7.9) |
| Chen et al., 2017 | Jakarta  (Indonesia) | 20 | **Healthy subjects:**  8 males and 12 females | 61.8 ± 11.54 | Morning | **ELISA kit:**  BioVision | **BDNF (ng/mL):** 0.0774 ± 0.0477 |
| Antunes-Lopes et al., 2013 | Porto (Portugal) | 40 | **Healthy subjects:**  20 males and 20 females | Males: 37.0 ± 12.3 (range 22-61); Females: 38.6 ± 13.4 (range 24-67) | Morning | **ELISA kit:**  Emax®ImmunoAssay System from Promega | **BDNF/Cr ratio (females):**  110.4 ± 159.5 (units N.R.) |
| Magalhães et al., 2017 | Minas  (Brazil) | 40 | **Healthy subjects:**  22 males and 18 females | From 28 to 32 incomplete weeks of gestational age | After birth, at 48h and 72h and at 3 weeks after birth. | **ELISA kit:**  R&D Systems | N.R. |
| Alkis et al., 2017 | Turkey | 45 | **Healthy subjects** | >18 | Urine was obtained from a full bladder. | **ELISA kit** | **BDNF/Cr (ng/ml):**  340.2 ± 199.0 |

N.R.: Not reported; BDNF: brain-derived neurotrophic factor; Cr: creatinine; ELISA: enzyme-linked immunosorbent assay.

**3.3. Application to INMA-Granada urine samples**


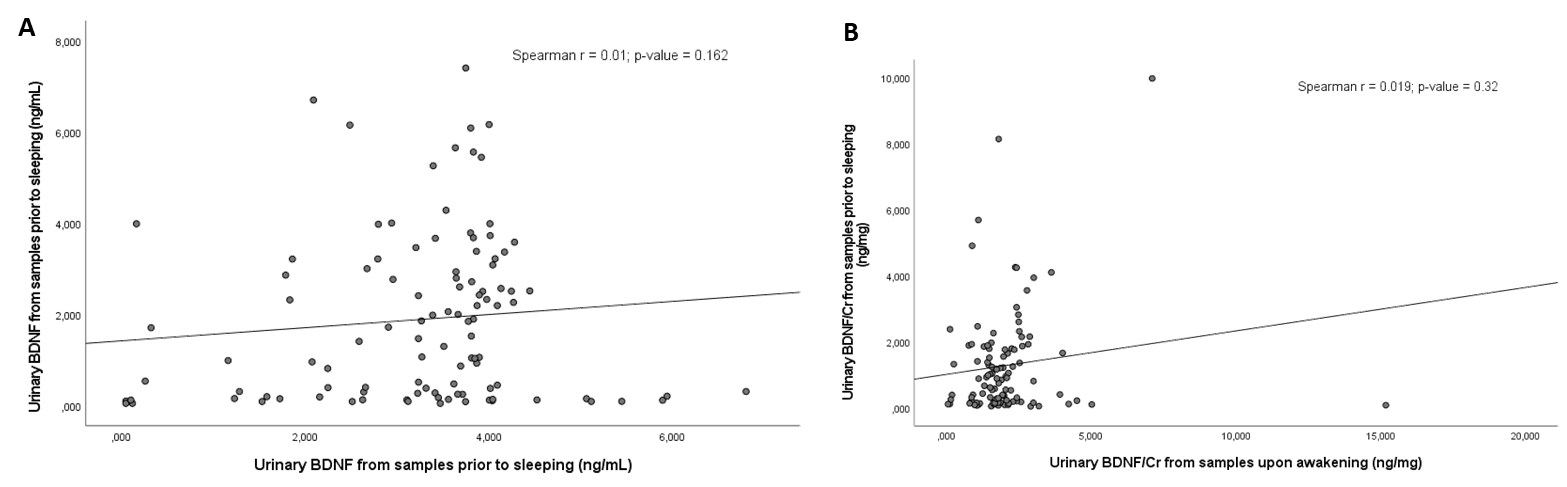


**Supplementary Figure 2**. Correlation of the urinary BDNF (A) and BDNF/Cr (B) concentrations obtained from samples upon awakening and samples prior to sleeping of the male adolescents (n=106).

**INMA-Granada Cohort serum samples:** Peripheral venous blood samples were drawn from participants under non-fasting conditions (5 - 7 p.m.) on the same day as the collection of the urine samples. Samples were immediately processed to obtain serum, which was subsequently stored at −80 C◦. Samples were defrosted for the analysis, vortexed, aliquoted in 10 μL, and diluted 100-fold. Total serum BDNF concentrations were measured with an enzyme-linked immunosorbent assay using the commercial Quantikine® ELISA kit (R&D Systems, Minneapolis, MN, USA) following the protocol and recommendations of the manufacture at the Biomedical Research Center (CIBM), Granada, Spain. Serum total BDNF protein concentrations had intra- and inter-assay CVs of <5% and 15%, respectively.


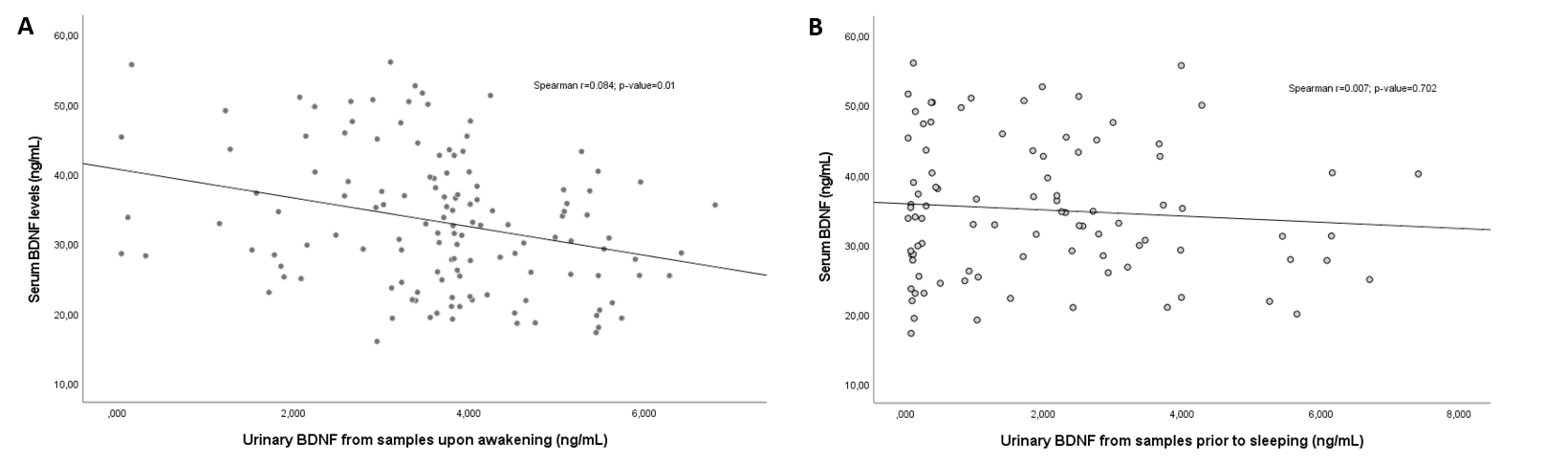


**Supplementary Figure 3.** Correlation of the serum BDNF (ng/mL) and urinary BDNF (ng/mL) concentrations obtained from samples upon awakening (n=132) and samples prior to sleeping (n=92) from the male adolescents.

**References (Table S2)**

Aisa, M. C., Barbati, A., Cappuccini, B., De Rosa, F., Gerli, S., Clerici, G., et al. (2021). Urinary Nerve Growth Factor in full-term, preterm and intra uterine growth restriction neonates: Association with brain growth at 30–40 days of postnatal period and with neuro-development outcome at two years. A pilot study. *Neurosci. Lett.* 741, 135459. doi: 10.1016/j.neulet.2020.135459.

Alkis, O., Zumrutbas, A. E., Toktas, C., Aybek, H., and Aybek, Z. (2017). The use of biomarkers in the Diagnosis and Treatment of Overactive Bladder: Can we predict the patients who will be resistant to treatment? *Neurourol. Urodyn.* 36, 390–393. doi: 10.1002/nau.

Antunes-Lopes, T., Coelho, A., Pinto, R., Barros, S. C., Cruz, C. D., Cruz, F., et al. (2017). Urinary Neurotrophin Levels Increase in Women With Stress Urinary Incontinence After a Midurethral Sling Procedure. *Urology* 99, 49–56. doi: 10.1016/j.urology.2016.08.048.

Antunes-Lopes, T., Pinto, R., Barros, S. C., Botelho, F., Silva, C. M., Cruz, C. D., et al. (2013). Urinary neurotrophic factors in healthy individuals and patients with overactive bladder. *J. Urol.* 189, 359–365. doi: 10.1016/j.juro.2012.08.187.

Chen, S. F., Jiang, Y. H., and Kuo, H. C. (2017). Urinary biomarkers in patients with detrusor underactivity with and without bladder function recovery. *Int. Urol. Nephrol.* 49, 1763–1770. doi: 10.1007/s11255-017-1666-z.

Collins, L. R., and Koven, N. S. (2014). Urinary BDNF-to-creatinine ratio is associated with aerobic fitness. *Neurosci. Lett.* 559, 169–173. doi: 10.1016/j.neulet.2013.12.004.

Ece, A., Coşkun, S., Şahin, C., Tan, Karabel, D., and Çim, A. (2019). BDNF and NGF gene polymorphisms and urine BDNF–NGF levels in children with primary monosymptomatic nocturnal enuresis. *J. Pediatr. Urol.* 15, 255.e1-255.e7. doi: 10.1016/j.jpurol.2019.03.010.

Ghoniem, G., Faruqui, N., Elmissiry, M., Mahdy, A., Abdelwahab, H., Oommen, M., et al. (2011). Differential profile analysis of urinary cytokines in patients with overactive bladder. *Int. Urogynecol. J.* 22, 953–961. doi: 10.1007/s00192-011-1401-8.

Jiang, Y. H., Jhang, J. F., Ho, H. C., Hsu, Y. H., and Kuo, H. C. (2022). Diagnostic and prognostic value of urine biomarkers among women with dysfunctional voiding. *Sci. Rep.* 12, 1–8. doi: 10.1038/s41598-022-10696-w.

Jiang, Y. H., Liu, H. T., and Kuo, H. C. (2014). Decrease of urinary nerve growth factor but not brain-derived neurotrophic factor in patients with interstitial cystitis/bladder pain syndrome treated with hyaluronic acid. *PLoS One* 9, 1–6. doi: 10.1371/journal.pone.0091609.

Koven, N. S., and Collins, L. R. (2014). Urinary brain-derived neurotrophic factor as a biomarker of executive functioning. *Neuropsychobiology* 69, 227–234. doi: 10.1159/000362242.

Kurku, H., Soran, M., Yar, A., Akdam, N., Arslan, Ş., and Gönen, M. (2019). Serum and urinary levels of brain-derived neurotrophic factor (BDNF) in enuresis. *Arch. Esp. Urol.* 72, 1032–1037.

Magalhães, R. C., Moreira, J. M., Vieira, É. L. M., Rocha, N. P., Miranda, D. M., and Simões E Silva, A. C. (2017). Urinary Levels of IL-1β and GDNF in Preterm Neonates as Potential Biomarkers of Motor Development: A Prospective Study. *Mediators Inflamm.* 2017. doi: 10.1155/2017/8201423.

March, B., Lockhart, K. R., Faulkner, S., Smolny, M., Rush, R., and Hondermarck, H. (2021). ELISA-based quantification of neurotrophic growth factors in urine from prostate cancer patients. *FASEB BioAdvances* 3, 888–896. doi: 10.1096/fba.2021-00085.

Morizawa, Y., Aoki, K., Iemura, Y., Hori, S., Gotoh, D., Fukui, S., et al. (2019). Urinary nerve growth factor can predict therapeutic efficacy in children with monosymptomatic nocturnal enuresis. *Neurourol. Urodyn.* 38, 2311–2317. doi: 10.1002/nau.24142.

Ozdemir, K., Dincel, N., Berdeli, A., and Mir, S. (2016). Can urinary nerve growth factor and brain-derived neurotrophic factor be used in the diagnosis and follow-up of voiding dysfunction in children? *Urol. J.* 13, 2690–2696. doi: 10.22037/uj.v13i3.3262.

Pennycuff, J. F., Schutte, S. C., Hudson, C. O., Karp, D. R., Malykhina, A. P., and M., N. G. (2017). Urinary Neurotrophic Peptides in Postmenopausal Women With and Without Overactive Bladder. *Neurourol. Urodyn.* 36, 740–744. doi: 10.1002/nau.

Philippova, E. S., Bazhenov, I. V., Ziryanov, A. V., and Bazarny, V. V. (2021). Impact of intradetrusor botulinum toxin A injections on serum and urinary concentrations of nerve growth factor and brain-derived neurotrophic factor in patients with multiple sclerosis and neurogenic detrusor overactivity. *Neurourol. Urodyn.* 40, 95–101. doi: 10.1002/nau.24534.

Rada, M. P., Ciortea, R., Măluţan, A. M., Doumouchtsis, S. K., Bucuri, C. E., Clim, A., et al. (2020). The profile of urinary biomarkers in overactive bladder. *Neurourol. Urodyn.* 39, 2305–2313. doi: 10.1002/nau.24487.

Wang, L. W., Han, X. M., Chen, C. H., Ma, Y., and Hai, B. (2014). Urinary brain-derived neurotrophic factor: A potential biomarker for objective diagnosis of overactive bladder. *Int. Urol. Nephrol.* 46, 341–347. doi: 10.1007/s11255-013-0540-x.

Wang, L. W, Li, J. L, Yu, Y., Xiao, R. H, Huang, H., Kuang, R. R, et al. (2017). Association of increased urine brain derived neurotrophic factor with lower urinary tract symptoms in men with benign prostatic hyperplasia. *J. Huazhong Univ. Sci. Technol. - Med. Sci.* 37, 531–535. doi: 10.1007/s11596-017-1768-y.
